# A novel, highly sensitive and specific biomarker for Niemann-Pick type C1 disease

**DOI:** 10.1186/s13023-015-0274-1

**Published:** 2015-06-17

**Authors:** Anne-Katrin Giese, Hermann Mascher, Ulrike Grittner, Sabrina Eichler, Guido Kramp, Jan Lukas, Danielle te Vruchte, Nada Al Eisa, Mario Cortina-Borja, Forbes D Porter, Frances M Platt, Arndt Rolfs

**Affiliations:** Albrecht-Kossel-Institute for Neuroregeneration, Medical University of Rostock, Gehlsheimer Str. 20, 18147 Rostock, Germany; PharmAnalyt Labor GmbH, Ferdinand-Pichler Gasse 2, 2500 Baden, Austria; Department for Biostatistics and Clinical Epidemiology, Charité-University Medical Centre, Berlin, Germany; Centogene AG, Schillingallee 68, 18055 Rostock, Germany; Department of Pharmacology, University of Oxford, Mansfield Road, Oxford, OX1 3QT UK; Population, Policy and Practice Programme, Institute of Child Health, University College London, 30 Guilford Street, London, WC1N 1EH UK; Eunice Kennedy Shriver National Institute of Child Health and Development, National Institutes of Health, 10 Center Drive, Bethesda, MD 20892 USA

**Keywords:** Niemann-Pick type C1 disease, NPC1, Biomarker, HPLC-MS/MS

## Abstract

**Background:**

Lysosomal storage disorders (LSDs), are a heterogeneous group of rare disorders caused by defects in genes encoding for proteins involved in the lysosomal degradation of macromolecules. They occur at a frequency of about 1 in 5,000 live births, though recent neonatal screening suggests a higher incidence. New treatment options for LSDs demand a rapid, early diagnosis of LSDs if maximal clinical benefit is to be achieved.

**Methods:**

Here, we describe a novel, highly specific and sensitive biomarker for Niemann-Pick Type C disease type 1 (NPC1), lyso-sphingomyelin-509. We cross-validate this biomarker with cholestane-3β,5α,6β-triol and relative lysosomal volume. The primary cohort for establishment of the biomarker contained 135 NPC1 patients, 66 NPC1 carriers, 241 patients with other LSDs and 46 healthy controls.

**Results:**

With a sensitivity of 100.0% and specificity of 91.0% a cut-off of 1.4 ng/ml was established. Comparison with cholestane-3β,5α,6β-triol and relative acidic compartment volume measurements were carried out with a subset of 125 subjects. Both cholestane-3β,5α,6β-triol and lyso-Sphingomyelin-509 were sufficient in establishing the diagnosis of NPC1 and correlated with disease severity.

**Conclusion:**

In summary, we have established a new biomarker for the diagnosis of NPC1, and further studies will be conducted to assess correlation to disease progress and monitoring treatment.

**Electronic supplementary material:**

The online version of this article (doi:10.1186/s13023-015-0274-1) contains supplementary material, which is available to authorized users.

## Background

Lysosomal storage disorders (LSDs) are a group of over 70 different inborn errors of metabolism with a combined incidence around 1 in 5,000 live births [1]. Symptoms depend on tissue distribution and function of the accumulating substrates [2]. Niemann-Pick Type C disease (NPC) is an autosomal-recessive LSD, that is reported to affect 1:120,000 live births [3], though the relative frequency in at-risk cohorts such as patients with neurologic and psychiatric symptoms, is reported to be as high as 1.2% [4]. In 95% of cases NPC is caused by mutations in the NPC1 gene (OMIM: *607623), only in 5% of cases have mutations in the NPC2 gene been reported (OMIM: *601015). Currently, Miglustat is the only EMEA-approved drug for the treatment of NPC [5,6], though novel approaches such as cyclodextrin [7] and histone-deacetylase inhibitors [8] are being investigated. Despite clinical evaluation of patients new clinical studies will also require assessment of independent, objective surrogate parameters to evaluate disease progression and response to treatment. In addition to the classical Filipin staining test, which is not always conclusive in patients with NPC [9], two biomarkers for NPC have been reported to date, oxysterols [10] and measurement of intracellular acidic compartment volume (mean equivalent of fluorescence - MEFL/LysoTracker) [11]. While oxysterols facilitate the primary diagnosis of NPC, MEFL is mainly useful in assessing the follow-up and treatment response of patients.

Here, we report the establishment of a novel biomarker for NPC1 and cross-validate our findings with published cholestane-3β,5α,6β-triol and MEFL data analyzing the same plasma data [11]. As a first step we screened ten NPC1 patients and 10 age- and gender-matched healthy controls utilizing HPLC and tandem mass spectrometry and carefully assessed differences in mass spectra. This resulted in the detection of lyso-sphingomyelin-509 (lyso-SM-509) as a potential biomarker for NPC. In the following step, we screened 135 NPC1 patients, 66 NPC1 carriers, 241 patients with other LSDs and 46 healthy controls for lyso-SM-509. The structure is very similar to lyso-sphingomyelin (lyso-SM), the chemical formula being (C_24_H_49_N_2_O_7_P), though so far we were not able to elucidate the exact structure of lyso-SM-509. In keeping with this finding, lyso-SM-509 is also highly elevated in patients with NP-A/B, though the disease is easier to diagnose due to availability of enzyme activity assays for sphingomyelinase.

In a second line assessment we compared lyso-SM-509 measurements with those published for cholestane-3β,5α,6β-triol and MEFL. Here, we report on the result of the establishment of lyso-SM-509 as a biomarker and the cross-validation with the previously published biomarkers.

## Methods

### Patients and blood samples

Blood samples were obtained from patients enrolled in the “Biomarker for Niemann-Pick Type C Disease (BioNPC)” trial (ClinicalTrials.gov identifier: NCT01306604) and underwent biochemical analysis or genetic testing for verification of a suspected metabolic disease by the Albrecht-Kossel-Institute for Neuroregeneration (AKos). Additionally, blood plasma samples collected in the scope of the investigation of relative acidic compartment volume in NPC have been analysed for retrospectively for lyso-sphingomyelin-509 levels [11].

### Genetic analysis, Oxysterol and LysoTracker measurements

Standard analysis of the NPC1 gene was performed according to standard protocols [12]. All measurements for cholestane-3β,5α,6β-triol and LysoTracker have been performed previously [11].

### Identification of lyso-sphingomyelin-509 as a biomarker for NPC

Screening for potential biomarkers was carried out using HPLC-MS/MS of blood plasma samples of 10 healthy subjects and 10 NPC1 patients with accurate mass MS-systems (Orbitrap XL) and tandem MS systems. Differences between NPC1 patients and healthy controls were carefully assessed with regards to substances strongly increased only in patients. Molecular weight and structure of such substances were determined carefully. Peaks were identified by accurate mass MS-system and measured by tandem MS/MS systems using already known internal standards as reference. After validating the robustness of lyso-SM-509, measurements in healthy controls, NPC1 carriers, NPC1 patients and in patients with other LSDs ensued (For details see Additional file 1: Figure S1 and S2). Chemical formula determination was done on an LTQ Orbitrap XL, parent ion as well as fragment ions were determined with accurate mass and high resolution leading to the chemical formula described. Purification steps were performed in order to receive larger and purer compound (SPE and liquid-liquid extraction steps-for purification only) to gain higher concentrated compound for HPLC determination and infusion experiments. Sodium aduct ions as well as negatively charged ions of the substance had also been determined and studied more in depth on a Q/TOF system. Stress tests of the substance in plasma had been performed, it appears that the substance is stable in plasma over several hours at room temperature, furthermore strongly acidic or alkaline conditions did not show significant degradation. Also several freeze/thaw cycles in human plasma showed stability of the compound.

### Method for determination of free lyso-sphingomyelin and lyso-sphingomyelin-509 in plasma

50 μL of the sample were mixed with 100 μL of Internal Standard (lyso-Gb2 (Matreya LLC, USA)) working solution (in EtOH) and were then mixed subsequently using a DVX-2500 Multi-tube vortex device at 2500 rpm for about 30 seconds. After centrifugation at 4000 rpm for 2 minutes the clear supernatant was transferred into auto-sampler vials and injected into the HPLC-MS/MS system. Mobile phase used for gradient elution was 50 mM formic acid in water and 50 mM formic acid in acetonitrile/acetone (1/1, v/v). HPLC flow was set at 0.9 mL/min on an ACE 3 C8 column (50 × 2.1 mm) at 60°C, the injection volume used was 5 μL. Retention time for the analytes were approximately 3.2 minutes for lyso-SM and 3.6 minutes for lyso-SM-509, and for the internal standard (lyso-Gb2 (Matreya LLC, USA)) approximately 3.2 minutes. Lyso-Gb2 was checked in regards to native concentrations in plasma and was found to be at very low levels only, a sufficient amount was added therefore during sample preparation. The MS/MS system used was an API 4000 using electrospray ionization in MRM mode in positive mode at 500°C for determination of free Lyso-Sphingomyelin in plasma. Quadrupole resolution was set at unit /unit, MRM transitions used were 465 → 184 m/z for the analyte, 509 → 184 m/z for 509, and 624 → 282 for the internal standard. Calibration was done from 2 ng/mL to 200 ng/mL in aqueous/methanolic solution; QC samples were spiked in plasma at levels of 15 and 150 ng/mL.

### Statistics

#### Statistics for lyso-sphingomyelin-509

Data were aggregated by taking the first measured value according to genotype (NPC1 patients, NPC1 carriers, NP- A/B patients, P-A/B carriers and healthy controls). Data is given in median and interquartile range where indicated. In order to analyze the diagnostic value of lyso-sphingomyelin-509 a receiver operating characteristic (ROC) curve was employed and the area under the curve (AUC) with 95%CI was calculated. Descriptive statistics and ROC curve analysis were done using SPSS Release Version 22 (© SPSS, Inc., 2013, Chicago, IL, www.spss.com).

### For comparison with MEFL and cholestane-3β,5α,6β-triol data

Logistic regression models were fitted to compare proportions, Wilcoxon-Mann-Whitney and Kruskal-Wallis tests were used to compare medians, and ANOVA was used to compare means where appropriate. Linear regression models were fitted with maximum likelihood. A locally adaptive super smoother was used to extract the data structure without a regression model [13]. These calculations were performed in the R language and environment for statistical computing (version 2.14.2; http://www.R-project.org). Graphs with error bars represent mean ± SD. A *p-*value smaller than 0.05 was considered significant.

### Study approval

The protocol of the BioNPC trial has been approved by the Research Ethics Committee of the University Rostock (ClinicalTrials.gov identifier: NCT01306604). Patients undergoing therapy were treated according to standard protocols. Written informed consent was obtained from all participants or their legal guardians.

## Results and discussion

### Establishment of lyso-sphingomyelin-509 as a biomarker for NPC and characterization of the study cohort

The recently emerging novel treatment approaches for treating NPC including cyclodextrin [7], HSP70 and HDAC inhibitors [8] strengthen the need for early diagnosis and monitoring of disease progression. Biomarkers for LSDs have been investigated for some time to facilitate diagnosis and monitoring response to therapy. In, 1989 Rosengren and colleagues investigated the glycosphingolipid pattern in late infantile metachromatic leukodystrophy (MLD) [14]. Lysosulfatide had been identified in MLD and normal brain tissue subsequently, the authors arguing for a de novo synthesis of lysoglycosphingolipids from sphingosine and arrive at the conclusion that they do not play a role in disease pathology [14].

Since then the perception of lysoglycosphingolipids has changed dramatically. For example several lysosphingolipid biomarkers having been established recently, e.g. lyso-Globotriaosylceramide (lyso-Gb3) in Fabry disease [15], Glucosylsphingosine (lyso-GL-1) in Gaucher disease [16], Galactosylsphingosine (Psychosine) in Krabbe disease [17,18] and lyso-sphingomyelin in Niemann-Pick B patients [19].

**Figure 1:**
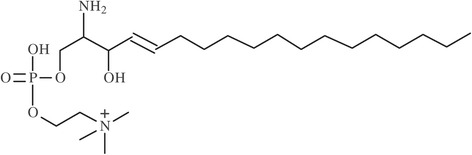
Chemical structure of Lyso-Sphingomyelin-509. This substance was analysed by HPLC-MS/MS with high sensitivity and specificity of diagnosing NPC. The precise structure of lyso-SM-509 has not yet been elucidated.

Here, we investigated a potential biomarker for NPC1, lyso-SM-509. For the establishment of a biomarker for NPC1 the plasma samples of 10 NPC1 patients and 10 healthy controls were analyzed by tandem mass spectrometry as described in the Material and Methods section. After initial testing, lyso-SM-509 (Figure 1, for additional information see Supplement) was determined as an interesting target for the primary diagnosis of NPC1. Though we have established C_24_H_49_N_2_O_7_P as the molecular formula for lyso-SM-509 (lyso-SM with the addition of carbon dioxide, the compound was dubbed for the mass weight examined, Figure 1), the exact structure is still subject of investigation. Current results suggest that the carbon dioxide is most likely located at the amino group of the lyso-SM (data not shown). A calculation of peak area ratio to internal standard was used for lyso-SM-509. To date, the mechanism by which lysoglycosphingolipids arise has not been elucidated, the most prominent hypothesis, also for lyso-Gb3 and lyso-GL1, being the deacylation of the corresponding glycosphingolipid [15,16,20].

**Figure 2:**
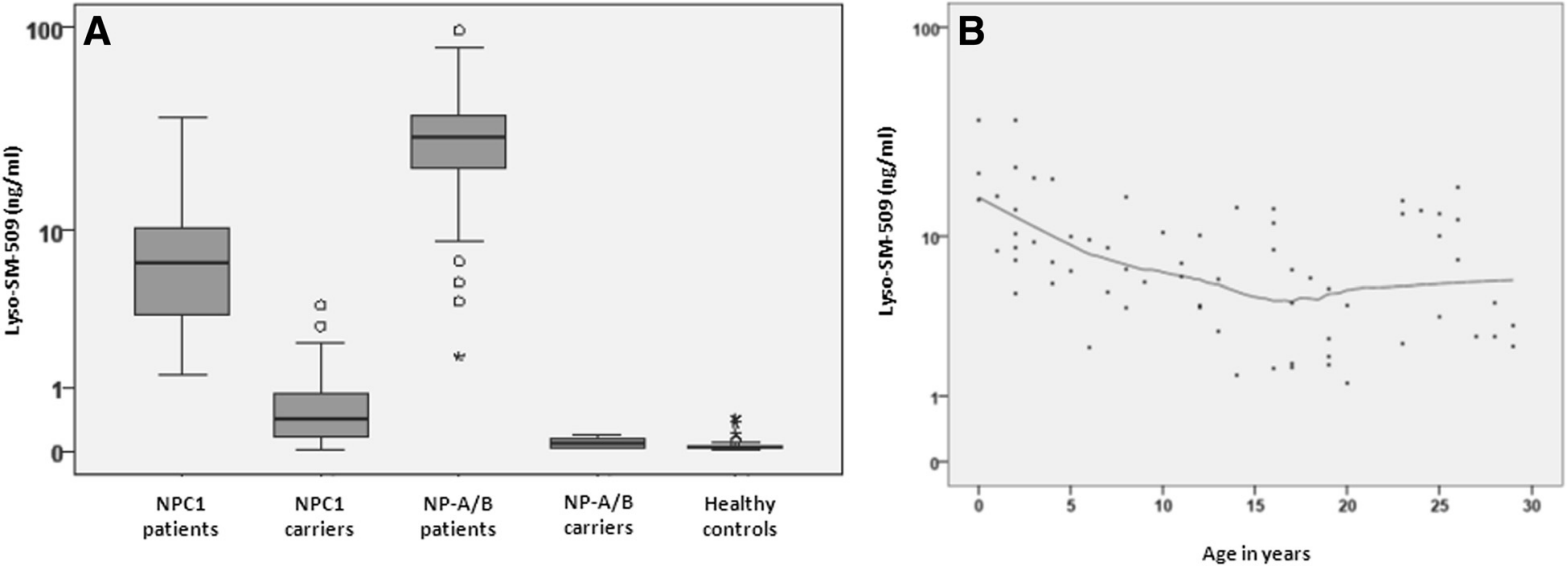
Levels of lyso-SM-509 in patients with NPC1 (n = 110), NP-A/B (n = 21), as well as healthy controls (n = 43) and NPC1 carriers (n = 63) and NP-A/B carriers (n = 5). **(A)** Lyso-SM-509 allows distinguishing between healthy controls and patients with NPC1. Notably, the biomarker levels are for NP-A/B patients were 3.55-times higher (29.4 ng/ml (IQR: 14.8-40.0). Notably, only in two cases lyso-SM-509 levels above 2.5 ng/ml can be detected in a carrier of a NPC1 mutation and in none of the healthy controls. **(B)** Levels of lyso-SM-509 in NPC1 by age. Note that Lyso-SM-509 is higher in younger NPC1 patients (*ρ* = -0.519, *p* < 0.001), reflecting the severity of early onset disease as shown by Te Vruchte and colleagues (11).

Overall, 242 subjects with originally 408 measurement points were analyzed, the main cohorts were (1) NPC1 patients (*n* = 110, median age 13 years, (2) NPC1 carriers (*n* = 63, median age: 46, (3) NP-A/B patients (*n* = 21, median age: 9, (4) NP-A/B carriers (*n* = 5, median age: 7), and (5) healthy controls (*n* = 43, median age: 41 (for details see Table 1). For NPC1 patients a median lyso-SM-509 value of 6.7 ng/ml was measured, NP-A/B patients displayed about 4-times higher values (29.4 ng/ml), while neither NPC1 carriers, NP-A/B carriers, nor healthy controls displayed elevated values (for details see Figure 2A, B and Table 2). Of note, in two instances NPC1 carriers displayed measurements above 2.5 ng/ml. Each were parents of genetically confirmed NPC1 patients and both underwent genotyping confirming their carrier status. In summary, a cut-point of 1.4 ng/ml for lyso-SM-509 was determined to distinguish NPC1 patients from NP-A/B patients, NPC1 carriers, NP-A/B carriers and healthy controls (sensitivity: 100.0%, specificity: 91.0%, ROC-curve analysis: Area under the curve (AUC): 0.99, 95% CI: 0.98-1.00) (Table 3). We conclude that lyso-SM-509 is useful in determining the primary diagnosis of NPC1.

**Table 1:** **Characteristics of investigated populations**

**Cohort**	**Individuals (** ***n*** **)**	**Measurements (** ***n*** **)**	**Age (first value)**	**Males (** ***n*** **)**	**Females (** ***n*** **)**
			**(median, IQR, number of cases)**	**Age (median, IQR)**	**Age (median, IQR)**
NPC1 patients	110	241	13 (4-20)	33	33
			(*n* = 67)	13 (4-19)	12 (5-23)
NPC1 carriers	63	94	46 (25-54)	8	12
			(*n* = 20)	48 (27-56)	45 (14-53)
NP-A/B patients	21	21	9 (1-15)	10	4
			(*n* = 14)	7 (1-15)	11 (3-47)
NP-A/B carriers	5	5	7 (min-max: 2-35)	1	2
			(*n* = 3)	7 yrs	Med.: 19
Healthy controls	43	47	41 (20-50)	3	9
			(*n* = 12)	51 (min-max: 4-71))	38 (21-46)

Individual level data on age and gender was not documented for all samples. Age and gender are stated only where available.

**Table 2:** **Medians and interquartile ranges for lyso-SM-509 in ng/ml for all investigated sub-cohorts**

	***n***	**Lyso-SM-509 in ng/ml**
NPC1 patients	110	6.7 (3.4-10.3)
NPC1 carriers	63	0.4 (0.2-0.9)
NP- A/B patients	21	29.4 (14.8-40.0)
NP- A/B carrier	5	0.09 (0.04-0.16)
Healthy controls	43	0.04 (0.03-0.06)

**Table 3:** **Sensitivity, specificity and accuracy of lyso-SM-509 in NPC1 patients**

	**Lyso-SM-509**
**NPC1 patients (** ***n*** **)**	110 (221)
Cut point/sensitivity range (ng/ml)	1.4
Sensitivity	100.0%
Specificity	91.0%
Accuracy	95.5%
AUC and 95%CI in ROC Analysis	0.99 (0.98-1.00)

### Relationship to MEFL and cholestane­3β,5α,6β‐triol

For NPC1, two other biomarkers have already been established – non-enzymatically generated Oxysterols and the LysoTracker (by assessing MEFL as a measure of relative lysosomal volume) [10,11]. In the second part of our paper, the newly established lyso-SM-509 has been compared to both in the same plasma samples. Even though, two different biomarkers have been established and can be utilized for different aspects of the disease (Oxysterols for primary diagnosis by measurement of either Cholestane-3β,5α,6β-triol or 7-ketocholesterol, which allows for discrimination between controls and NPC1 subjects [10] vs. MEFL for monitoring of disease progression and treatment response, which has been assessed in a 5-year prospective study [11]), an ideal biomarker for the primary diagnosis, monitoring of disease progression and treatment response has not yet been established. Lyso-sphingomyelin-509 as a biomarker for NPC adds discriminatory power to identify and will further facilitate early disease diagnosis. In fact, many diseases – even common diseases such as myocardial infarction – involve several biomarkers being used for primary diagnosis and follow-up. Therefore, our findings significantly contribute to the current literature on diagnosing NPC by adding a new biomarker.

**Figure 3:**
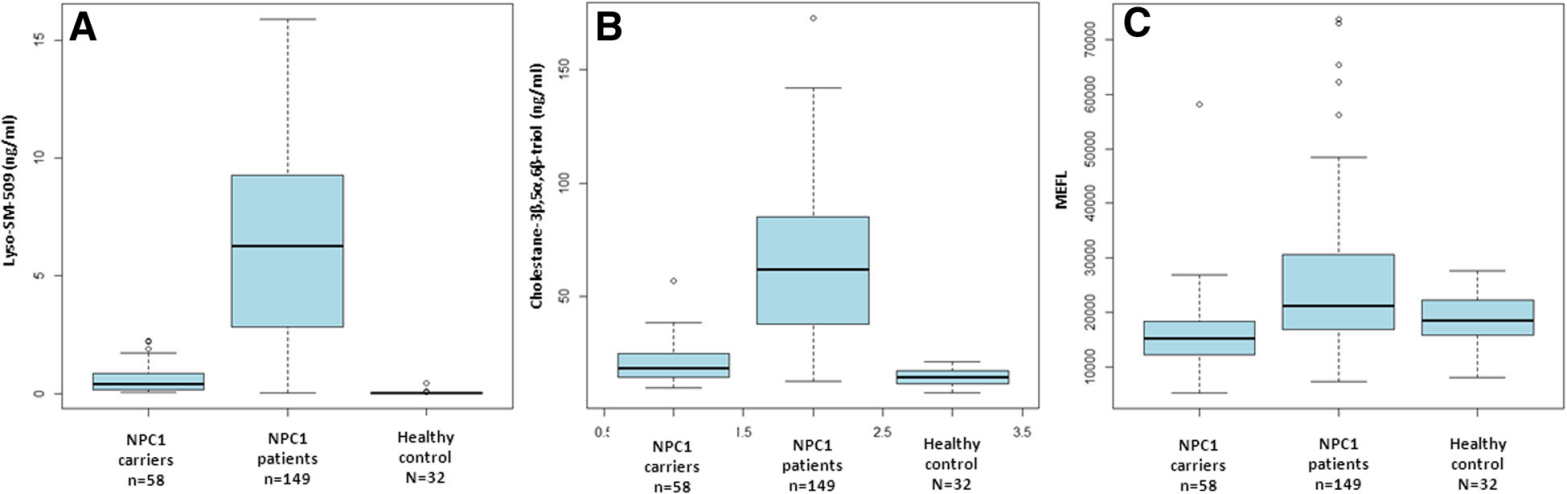
Comparison of Lyso-SM-509, cholestane-3β,5α,6β-triol and MEFL. **(A)** Boxplot of lyso-SM-509 by genotype, **(B)** boxplot of cholestane-3β,5α,6β-triol by genotype, **(C)** boxplot of MEFL (LysoTracker) by genotype. Both lyso-SM-509 and cholestane-3β,5α,6β-triol facilitate the distinction of NPC patients from healthy controls.

**Figure 4:**
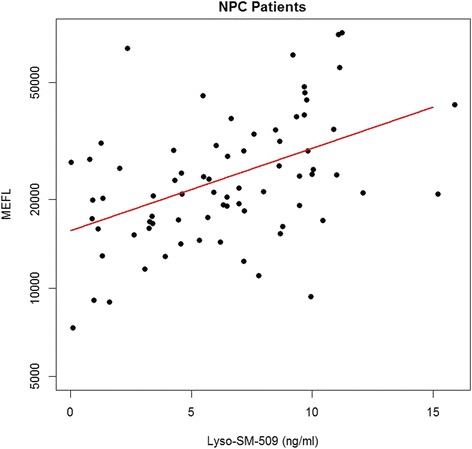
Comparison of MEFL (LysoTracker) (log scale) and lyso-SM-509 (linear scale). Levels of lyso-SM-509 significantly correlate with MEFL in NPC1 patients (*ρ* =0.432, *p < 0.001*). The Red line is a linear model.

**Figure 5:**
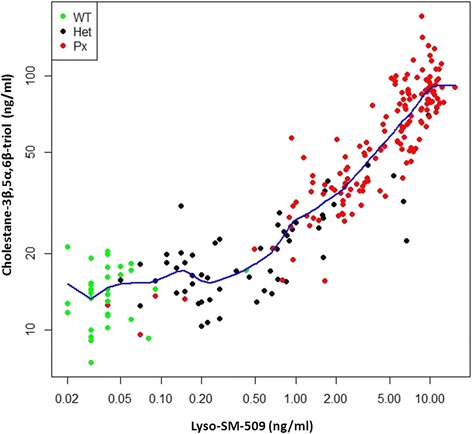
Cholestane-3β,5α,6β-triol vs lyso-SM-509 (log-log scale) by genotype. Lyso-SM-509 and cholestane-3β,5α,6β-triol correlate very well by genotype (Healthy controls, NPC1 carriers and NPC1 patients; *ρ* = 0.675, *p* <0.001); blue line is a super smoother.

In order to investigate the relationship of lyso-sphingomyelin-509 to MEFL and cholestane-3β,5α,6β-triol (Oxysterols), we measured its values in 240 samples from 125 subjects (NPC patients, NPC heterozygotes and healthy controls) that were previously collected and analyzed by FM Platt and colleagues in the scope of a 5-year international prospective study investigating lysosomal volume in B cells from NPC patients [11]. Data for this subcohort of samples are summarized in Figure 3A-C. Levels of lyso-SM-509 correlate significantly with MEFL in NPC1 patients; *ρ* denotes Spearman’s correlation coefficient (Figure 4, *ρ* = 0.432 *p < 0.001*). In addition, lyso-SM-509 levels correlate very well with cholestane-3β,5α,6β-triol (*ρ =* 0.921, *p* < 0.001) (Figure 5) that had been measured in the same samples and the values previously published [10], samples overlapping with controls (lyso-SM-509 < 2.5 ng/ml) were excluded for the analysis. Taken together, lyso-SM-509 correlated well with both cholestane-3β,5α,6β-triol and MEFL, though lyso-SM-509 and cholestane-3β,5α,6β-triol were very highly correlated, suggesting they may be generated at least in part by the same process. In line with this, the LysoTracker assay was found to be semi-independent of both biochemical markers and combining the MEFL with either one adding discriminatory power to the analysis.

### Correlation of biomarkers with severity scores

**Figure 6:**
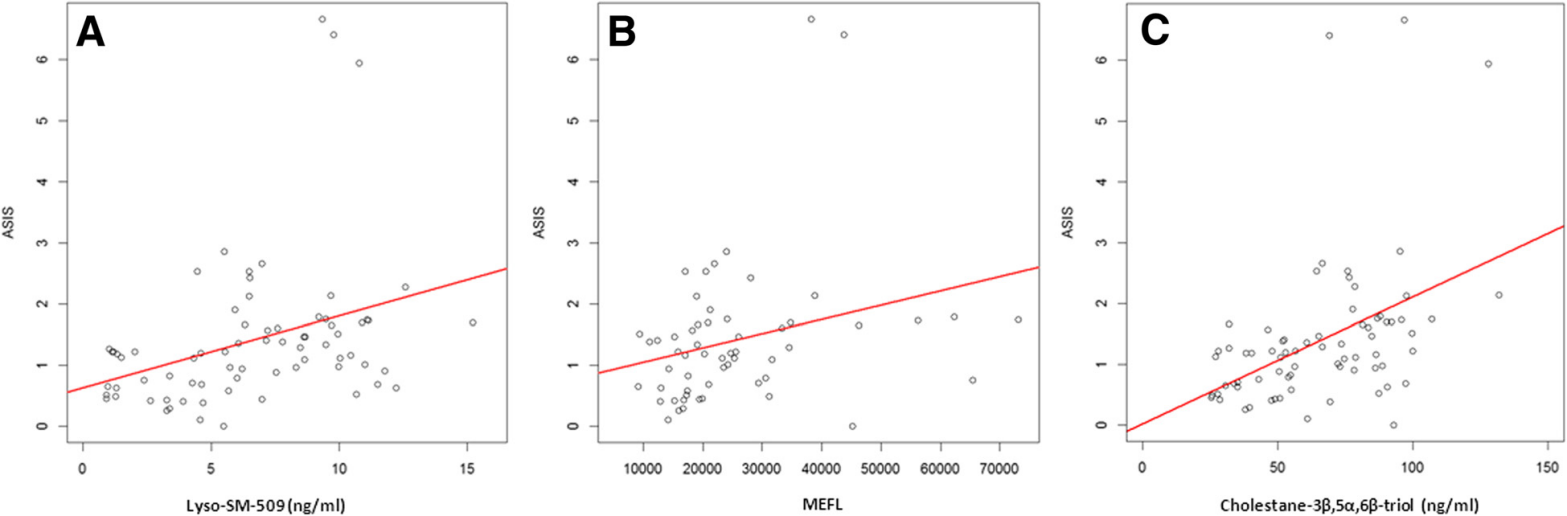
Linear Modelling for all NPC patients. Linear modelling was performed for all NPC patients were ASIS, Lyso-SM-509 **(A)**, MEFL **(B)** and cholestane-3β,5α,6β-triol **(C)** were available. Each patient contributes only one measurement. All three biomarkers significantly correlated with ASIS (*p* = 0.003 **(A)**, *p* = 0.039 **(B)**, and *p* < 0.001 **(C)**. The red lines correspond to the linear models fitted.

A randomly selected subgroup of 73 samples with measurements from 36 NPC1 patients for cholestane-3β,5α,6β-triol and lyso-SM-509 (median age: 12.76 (IQR: 9.83-17.28)) and MEFL with 57 measurements in 30 patients (median age: 13.00 (IQR: 10.38-18.49)) were analyzed for correlations with the total severity score of the patients (excluding hearing). In agreement with previous studies on the cholestane-3β,5α,6β-triol and MEFL there were no significant correlations with any of these biomarkers (data not shown) [10,11]. We also found no significant correlation between lyso-SM-509 and total severity score (*ρ* = 0.031, *p =* 0.794). However, as NPC1 is a progressive neurodegenerative disorder, and patients continue to progress over a number of years the annual severity increment score (ASIS) [11] was calculated by dividing the total severity score by age to determine relative rate of disease progression. As in the original NPC cohort study carried out by Frances Platt and colleagues [11], hearing was excluded since it was not measured in all centers. Lyso-SM-509 was significantly correlated to ASIS (slope *p* = 0.003), as were cholestane-3β,5α,6β-triol (slope *p* < 0.001) and MEFL (slope *p* = 0.039) (Figure 6). Analysis of the effect of therapy (miglustat treated vs untreated patients) was underpowered due to low numbers of untreated patients (*n*- = 21) so a larger study will be needed to address this.

## Conclusion

In summary, we have established a novel, sensitive and specific biomarker for the primary diagnosis of NPC1. The major advantage of lyso-SM-509 is that it is quick and easy to measure and standardize. As a consequence, lyso-SM-509 is definitely a useful biomarker for simple diagnostic in blood plasma, future investigations will analyze the sensitivity and specificity of lyso-SM-509 on dried blood spot samples, which would further simplify the diagnostic process.

## Additional file

Additional file 1: Figure S1. Chromatogram of lyso-SM-509 (top chromatogram) and the internal standard (bottom) in human control plasma. There is almost no lyso-SM-509 detectable in the control sample. **Figure S2.** Chromatogram of lyso-SM-509 (top chromatogram) and internal standard (bottom) in NPC1 patient sample at a high concentration.
