# Supplementary material for: A novel, highly sensitive and specific biomarker for Niemann-Pick type C1 disease
Source: Orphanet J Rare Dis. 2015 Jun 17;10:78. doi: 10.1186/s13023-015-0274-1 (PMC4479076; doi:10.1186/s13023-015-0274-1)
Supplement: Additional file 1: Figure S1. — Chromatogram of lyso-SM-509 (top chromatogram) and the internal standard (bottom) in human control plasma. There is almost no lyso-SM-509 detectable in the control sample. Figure S2. Chromatogram of lyso-SM-509 (top chromatogram) and internal standard (bottom) in NPC1 patient sample at a high concentration. [file 13023_2015_274_MOESM1_ESM.pdf]

Supplemental Figures:

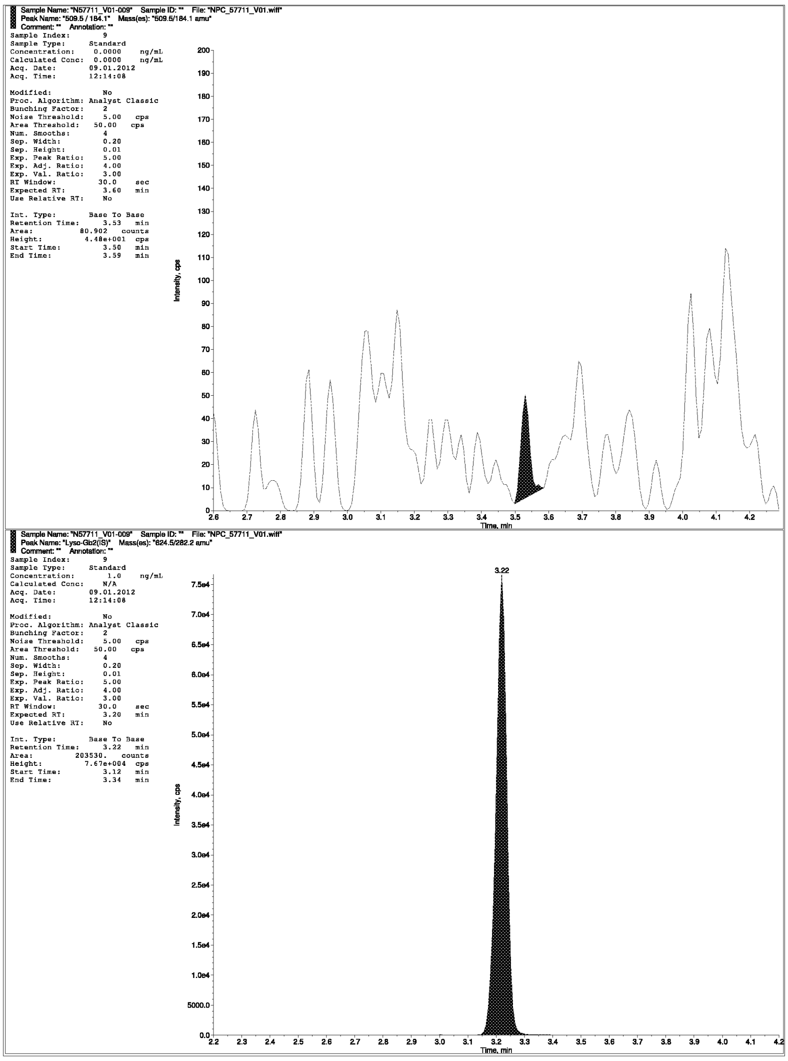

Figure S1: Chromatogram of lyso-SM-509 (top chromatogram) in human control plasma and the internal standard (bottom) calibrated at 6ng/ml. There is almost no lyso-SM-509 detectable in the control sample.

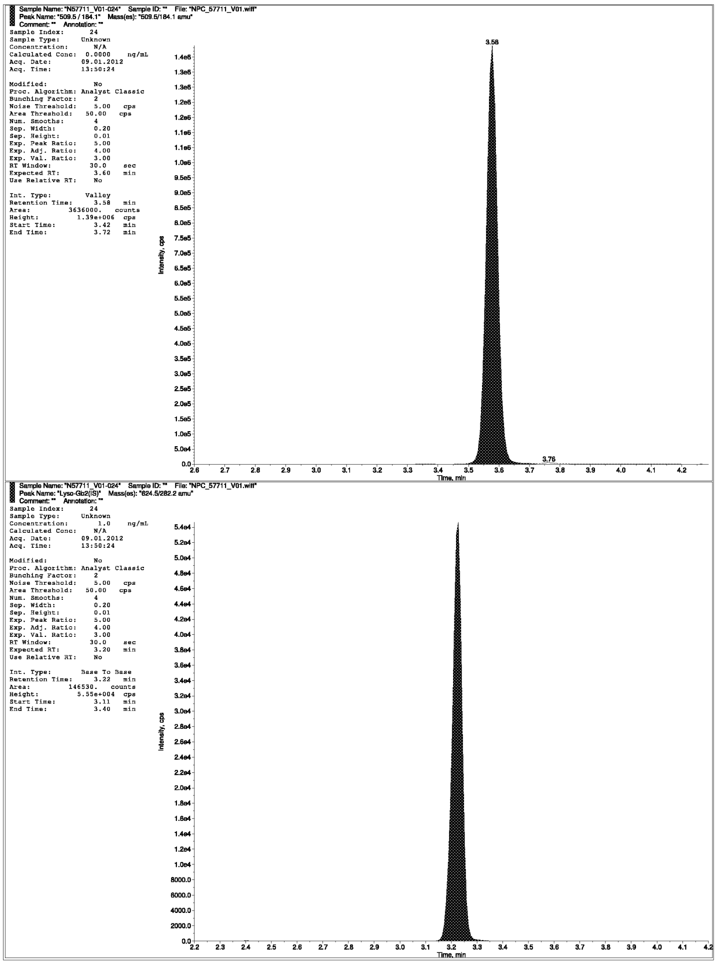

Figure S2: Chromatogram of lyso-SM-509 (top chromatogram) and internal standard (bottom) in NPC1 patient sample at a high concentration.
